# Chronic kidney disease and the alternative labels used by GPs in Australia: a qualitative interview study

**DOI:** 10.3399/BJGPO.2024.0031

**Published:** 2025-01-15

**Authors:** Michelle Guppy, Esther Joy Bowles, Paul Glasziou, Jenny Doust

**Affiliations:** 1 Institute for Evidence-Based Healthcare, Faculty of Health Sciences & Medicine, Bond University, Gold Coast, Australia; 2 School of Rural Medicine, University of New England, Armidale, Australia; 3 Institute for Evidence-Based Healthcare, Faculty of Health Sciences & Medicine, Bond University, Gold Coast, Australia; 4 Australian Women and Girls Health Research (AWaGHR) Centre, School of Public Health, Faculty of Medicine, The University of Queensland, Herston, Australia

**Keywords:** chronic kidney disease, renal insufficiency, chronic, ageing, aged, general practice

## Abstract

**Background:**

Guidelines for terminology defining chronic kidney disease (CKD) have been in use for 20 years. Age is not currently considered in the guideline definition of CKD. In previous studies, GPs have been reluctant to give older patients the label of CKD.

**Aim:**

To determine what language GPs are using to describe or label CKD with their older patients, and to explore the reasons for their use of alternative language.

**Design & setting:**

This was a descriptive qualitative interview study of Australian GPs.

**Method:**

Twenty-seven GPs were recruited via email and interviewed regarding their management of CKD. GPs were asked what language and terminology they used when discussing a diagnosis of CKD with their older patients.

**Results:**

'Labelling of CKD', the language that GPs use when talking about CKD with their patients, emerged as a major theme from the initial GP interviews. Sub-themes emerged, including types of alternative labels and rationale for alternative labels. GPs used descriptions of 'reduced kidney function' to explain CKD to their patients, either in parallel with the diagnosis of CKD or instead of it. GPs had concerns about the words 'chronic' and 'disease', and used different terminology to explain these words to patients when diagnosing them with CKD.

**Conclusion:**

GPs use alternative descriptions to explain mild decrease in kidney function with older patients. Alternative labels that denote level of risk to older patients, without creating unnecessary concern about normal age-related kidney function, need to be explored.

## How this fits in

GPs have been known to underuse the term 'chronic kidney disease' with their older patients with declining kidney function. This study describes the alternative terms that GPs prefer, and explains GPs’ rationale for using alternative terms with their patients.

## Introduction

Chronic kidney disease (CKD) is currently defined as '*abnormalities of kidney structure or function, present for greater than 3 months, with implications for health, and is classified based on cause, glomerular filtration rate (GFR) category and albuminuria category*'.^1^ This international definition was first introduced in 2002, bringing together a range of diseases into a framework of five stages of disease, with standardised nomenclature.^1^ The estimated glomerular filtration rate (eGFR) equation used in Australia is the CKD-Epidemiology Collaboration (CKD-EPI) equation. Quality improvement incentives to identify patients with disease started in the UK in 2006, including a CKD register.^2,3^ In Australia, guidelines for management of CKD in general practice were first published in 2007.^4^ The new guidelines brought together disparate language, including renal insufficiency, renal impairment, and renal failure into a consistent structured terminology.^5^

However, GPs have been identified as being slow to use this terminology, and reluctant to accept the national guidelines.^3,6^ GPs have raised concerns about labelling a patient with disease when their kidney function is only mildly impaired.^6^ This reluctance to give patients the label of CKD particularly arises in stage 3 CKD, with respect to an older population of asymptomatic patients.^2,3^ Kidney function is lower in older people, and is usually caused by physiological ageing.^7^ The current definition does not separate kidney disease from kidney ageing.^8^ Progression to end-stage kidney disease in older patients with mild kidney dysfunction and no proteinuria is rare.^8^ Half the older adult population aged >75 years meets the definition of CKD, and given the current demographic profile in Western countries, this forms a considerable portion of the population seen by GPs.^5^

Structured CKD terminology has now been recommended by international peak bodies for 20 years. At the time of this study (2019), guidelines had been in place in Australia for 12 years. We aimed to explore whether GP concerns around labelling were still occurring, and if they were, what terminology were GPs using to describe CKD in the older patient population. This qualitative study was part of a larger mixed-methods study on the management of CKD by GPs. We aimed to explore what language and labelling GPs used when discussing a diagnosis of CKD with their patients.

## Method

### Study setting

This study was a small qualitative component of a larger mixed-methods study. The larger study had the following three stages: a survey about GPs' knowledge, awareness and usage of CKD guidelines; a randomised vignette study on CKD diagnosis and management;^9,10^ and a qualitative component about GP management practices with respect to CKD. Participants were Australian GPs, originally recruited via an email sent by a third party publishing organisation, the Australasian Medical Publishing Company (AMPCo) asking them to participate in a survey about CKD. GPs who completed the survey were invited to participate in a further interview (Supplementary Box S1). GPs were purposively recruited to ensure variance in age, sex, and practice location, including rural practice, and distribution throughout Australia. Recruitment continued until saturation of themes was reached. Each GP interview took place in 2019, either face to face, or online via Zoom and were conducted by one researcher (MG) for up to 1-hour duration.

### Data collection and analysis

Data were collected through semi-structured interviews. Audio-recordings of the interviews were transcribed, and reviewed by interviewees for correction. Transcripts underwent reflexive thematic analysis,^11^ done independently by two researchers (MG and EJB). Codes were discussed and compared after several initial interviews, and then the remainder of the interviews coded independently. Emerging themes were reviewed against the study aims. The findings were presented to the broader research team for reflection and review. The researchers reflected on the data and reviewed and refined the themes to ensure alignment with the research questions and robustness to support the data. Decisions made during meetings were documented to ensure confirmability, dependability, and credibility.^12^ Participant quotes illustrate the findings, which multiple researchers reviewed.^13^ These strategies help to overcome researcher assumptions but, as argued by Gadamer,^14^ such assumptions are part of our understanding and hence researchers must recognise their personal beliefs, theories or assumptions as part of the analytical process. NVivo (version 12) was used to assist the thematic analysis.

## Results

Emails were sent to 9500 GP email addresses. There were 469 responses to the survey, of whom 400 provided demographic information (Supplementary Table S1). Eighty-three GPs agreed to participate in the qualitative component, of whom 27 GPs were interviewed. There were no dropouts. GP demographics can be seen in Table 1a, and a comparison with the general population of Australian GPs in Table 1b. Our sample contains a higher proportion of female GPs, and we oversampled GPs in rural and remote communities.

**Table 1.** (a) Demographics of GP interview participants (*n* = 27). (b) Demographics of Australian GPs in 2023 (*n* = 39 239)^26^

**(a) Age (years)**	31–40, 33%	41–50, 30%	51–60, 15%	61–70, 15%
**Sex**	Male, 40%	Female, 60%		
**Practice location**	Urban, 44%	Regional, 22%	Rural, 22%	Remote, 15%
**(b) Age (years)**	<39, 25%	40–54, 37%	55–64, 21%	65+, 17%
**Sex**	Male, 51%	Female, 49%		
**Practice location**	Urban 68%	Non-urban 32%		

^a^Two GPs did not state their age.

### Themes

'Labelling of CKD', the language that GPs use when talking about CKD with their patients, emerged as a major theme from the first six GP interviews. With the six initial GP interviews, the topic of labelling was not specifically explored, and it emerged as a theme as the interviews progressed. For the remaining 21 GP interviews, specific questions about labelling were added to the interview schedule (Supplementary files). Sub-themes that emerged included: GPs' use of the CKD terminology, rationalisation of alternative labelling, and types of alternative labels and language used.

#### Use of chronic kidney disease terminology

GP responses fell into three overlapping categories when asked about the language and terminology that they used when discussing a diagnosis of CKD with their older patients. Some always use the term 'chronic kidney disease' (*n* = 4), some never use CKD (*n* = 7), and some use a combination of CKD and other terminology (*n* = 10).

#### Rationale for alternative labels

Reasons for never using the term CKD with patients varied in the never-use group, with some GPs questioning the construct of the CKD diagnostic framework. Two GPs explained that CKD was new terminology as a rationale for not using it. Other GPs were more concerned about patient context and patient understanding. A GP who worked with Aboriginal patients never used the term CKD, but always annotated the diagnosis in the medical record as CKD. The rationale for this was that patients would automatically hear something else if the term CKD was used, which then set back their understanding and management. Some GPs had concerns with use of the word 'chronic' and some of the GPs did not use the word 'disease'. The rationale for this was that these words were difficult for patients to understand and were often misinterpreted by patients to mean severe disease.

For GPs in the mixed-usage group, many responded that they would first use lay language to describe what is going on, and then tell the patient that in medical terms this is called ‘chronic kidney disease’. The rationale is similar in that GPs recognised that significant explanation was required for patients first, in order for them to understand what the medical terminology means. They were concerned that otherwise patients might misunderstand the severity of their condition. Some GPs would describe CKD in the context of their patients’ other diagnoses, for example, diabetes or hypertension, to ensure understanding that the patient had not developed a new, separate disease.

GPs in the group who always and consistently use the term CKD described how they explain what this means to patients. Their rationale was consistent with the other groups in that they recognised the medical terminology needs a plain language explanation to aid patient understanding. One GP described the benefits of the term CKD, and was in favour of it in preference to older terminology, such as renal failure, explaining that CKD sounded less severe than the word ‘failure’. Another GP explained that it is important for patients to know their diagnosis, in case they are seeing other doctors who might use that terminology. (Table 2)

**Table 2.** GPs’ rationale for alternative labels

CKD as a new construct	*'I don't use the word chronic kidney disease because that’s a new term, only been in existence in the past 10 years or so, before there was no chronic kidney disease. So I tell them, look your kidneys are not working as well as they should, that’s how I tell them, we need to protect them because if there’s damage to the kidneys, you can't go back on it, so we need to protect your kidneys and then I tell them what things will damage the kidneys.*' (GP08)
Patient misunderstanding of terminology	*'I kind of think their take on that will be, oh I've got this awful terrible thing that is going to kill me, which it just sounds so bad.'* (GP25)
	*'I think chronic often means, for a lot of people it means severe and drastic and so it’s a very negative connotation and I think that generally I don't find it very helpful to say that.'* (GP50)
	*'It upsets patients and puts them into feeling anxious that there’s something wrong with them, so it directly impacts on their mental health. Disease is not a word I use when I'm talking to them about a diagnosis.'* (GP51)
Balance of lay and medical terminology to ensure understanding	*'I suppose you'd probably soften the blow and when you first start talking to them, you'll say, oh look your kidneys aren't great, they're not performing as well*.' (GP18)
	*'I'd say, oh look the medical term is CKD which means chronic kidney disease and I tell them, it’s just a word that we — it’s what it’s called but the way I like to think of it is, is that your kidneys aren't working as well.'* (GP36)
Ensure patient understanding of the context of kidney disease	*'Sometimes again it’s a case of we might be diagnosing diabetes at the next appointment, … and talking about kidneys as part of that.*' (GP36)
Rationale for always using the label 'CKD'	*'But I always explain to them, you've got chronic kidney disease, chronic means that it’s a longstanding disease, it’s been there for more than three months and it’s not going to go away.'* (GP75)
	*'I think chronic kidney disease sounds — will be my choice, it sounds better than what people use of renal failure or kidney failure. That word failure actually freak*[s] *out a lot patients, it’s just like the kidneys not working, you know? So chronic kidney disease give*[s] *them an impression that it’s something still treatable, manageable, there is a hope to change that because they've got a disease.' (GP44)*

CKD = chronic kidney disease

#### Types of alternative labels

Regardless of whether they used the term CKD, all GPs used other terminology to help explain to their patient what was happening with their kidneys. Most of the alternative language used by GPs focused on kidney function, and the reduction in performance of the kidneys with reference to normal functioning (see Table 3).

**Table 3.** Alternative labels used by GPs to explain chronic kidney disease

GP05	Kidney is not functioning well, renal impairment
GP08	Kidneys are not working as well as they should
GP10	Kidney problems, leaky kidneys
GP18	Your kidneys aren’t great
GP19	Your kidney is not coping well, not able to function on its optimal level
GP22	Your kidneys aren’t functioning quite as well as they should be
GP25	Your kidneys aren’t working very well, aren’t functioning as well as they could
GP30	Your kidneys are not functioning as well as they previously did in the past
GP32	Kidneys are not working as well as they used to do — as well as we would like them to
GP36	Your kidneys aren’t working as well as they should be, kidney impairment
GP41	Kidney problem, your kidneys aren’t functioning as well as they ought
GP44	Renal insufficiency, deteriorating kidney function
GP50	The kidneys aren’t functioning as well as when they were younger
GP51	A drop in kidney function or renal function, chronic kidney injury
GP57	Kidney dysfunction, (kidneys) are not working as efficiently as they could or should
GP61	Kidney damage
GP62	Renal failure or kidney failure
GP66	Kidneys aren’t working at 100%
GP69	Chronic kidney problem or chronic kidney issue
GP75	Your kidneys aren’t working as well as they should be

## Discussion

### Summary

While many GPs are now using the terminology of CKD with their patients, most are qualifying this and describing the disease as a reduction in function. Some GPs are still not using the terms 'chronic' or 'disease' with their patients, citing concern about creating unnecessary stigma and anxiety among their older patients with mild degrees of dysfunction.

### Strengths and limitations

The study was designed to sample a diverse range of GP views. In order to ensure rigour of our qualitative inquiry, we determined sampling adequacy by continuing to recruit GPs until we had saturation of themes and replication. This was an iterative approach as two researchers analysed the data and reviewed coding and themes concurrently, as the data were being collected in order to attain reliability and validity. Theoretical constructs were developed and tested as new data were collected as part of the iterative process.^15^ The qualitative study included the views and experiences of 27 GPs; a good sample for a qualitative study. The use of purposive sampling to ensure a representative sample of GPs may have biased the sample. GPs were recruited to this study via response to email, and we may have sampled GPs who had greater experience or interest in managing CKD. We believe, however, that ensuring a wide range of demographic characteristics in the sample has enriched the study outcome. GPs were interviewed from a variety of practice locations in urban, regional, rural and remote Australia, with a variety of ages and sex. The theme of labelling was discussed specifically with 21 GPs. GPs were very consistent in their responses with regard to alternative labelling of CKD. The findings of this study represent the experiences of GPs who volunteered to participate; other participants may have differing experiences. Our results may not be generalisable to other countries and healthcare systems; however, a comparison with the literature reveals concordant findings. Further studies with a different sampling approach are required in the future.

### Comparison with existing literature

Our results are consistent with previous studies performed in the UK, where GPs were reluctant to give patients the label of CKD, especially in stage 3 in an older population of asymptomatic patients.^2,3^ In these studies, GPs felt the CKD label had stigmatising effects, and could create overconcern about the risk of dialysis in the future.^2^ GPs reported a difficult balance between giving patients a potentially confusing diagnosis, and not wanting to conceal information.^16,17^ GPs in the UK reported that 'chronic' was often misinterpreted by patients to mean 'serious', and that kidney disease means dialysis or kidney transplant.^6^

Similar to our findings, in these UK studies the GPs also typically told their patients they had a problem with their kidney function.^6^ However, in the UK fewer patients were told they had a diagnosis of CKD.^6^ Labels used in these studies included 'borderline', 'under par', 'leaky kidneys', 'lack of wellness', or 'kidney strain'.^3,6,16^ Many GPs described reduced kidney function as part of the normal ageing process with accompanying reassurance to their patients.^3,16^ Similarly to our study, the kidneys were viewed as another parameter to be addressed (rather than a new disease) as part of managing diabetes or hypertension.^3^

The issue around what label to use is very important, because studies in the UK show that patients are often not told that they have a diagnosis of CKD. In one study in UK general practice, only 41% of patients were aware of their diagnosis of CKD, and they were more likely to be younger with a stage 3b or greater.^18^ In another US study, less than half of those with a high risk of progression to kidney failure knew they had a diagnosis of CKD, and less than 10% of low-risk people were aware of their diagnosis.^19^ Similarly, a recent study in ambulatory care in Germany reported only 9% of patients with CKD being aware of their diagnosis, and only 20% were coded in the medical record.^20^ There are important harms to consider for a patient who has not been told they have a problem with their kidney function, particularly around the use of medications, both prescribed and non-prescribed, and the risk of acute kidney injury.^21^ It is important to consider whether these patients have an understanding of their reduced kidney function even if they haven’t received a diagnosis of 'CKD', as this could have important outcomes in terms of prognosis and self-management. It is also important to understand what labels are used with increasing severity of reduced kidney function. GPs in our study reported that they would use ‘*stronger language’* in patients with stage 4 or 5 kidney disease, and be more concerned with unstable kidney function. Situations of patients at higher risk warrant a clear understanding of how labels affect patient understanding and patient management.

Labelling of CKD sits in the broader context of disease labelling of other conditions, where the burden of disease management lies within primary care.^22^ In addition, '*professional reticence to disclose CKD may reflect wider concerns that the proliferation of health-related discourses contributes to the medicalisation of individuals*'.^3^ CKD is not the only disease for which labelling has a problematic discourse. Concerns have been raised by GPs about other conditions being overmedicalised, including hypertension, non-alcoholic fatty liver disease, osteoporosis, obesity, psychiatric conditions, and normal ageing.^23^ Decline in kidney function is a normal part of the ageing process. Kidney function can decline in older age with no sign of kidney damage, and so reduced kidney function in older age can be considered physiological.^24^ More appropriate labelling of older patients with mild decline in kidney function is important to ensure patients understand their risks, but are also not unnecessarily harmed by overdiagnosis.

### Implications for research and practice

Many GPs report that the term CKD is confusing for older patients with mild dysfunction of the kidneys, and prefer to use terminology around 'reduced kidney function'. Would 'RKF' be a more widely accepted terminology, leading to better acceptance by GPs and older patients, while still recognising and managing patients’ risk factors? Further research should be done on utilising different terminology in general practice, and terms that would be acceptable to patients and GPs. A 'community jury' is one such research strategy that could be used to determine acceptable labels.^25^ Use of age-related percentile charts of kidney function to help GPs and patients recognise what is normal kidney function for different ages could be a useful strategy to determine when alternative terminology be used, rather than a diagnosis of CKD.^8–10^ 'Age-related reduced kidney function' could be another term to consider, reflecting the significant but natural decline in kidney function with age.

There are important harms to consider for an older patient who has not been told they have a problem with their kidney function. This is particularly around the use of medications, both prescribed and non-prescribed, and their potential risk of acute kidney injury.^21^ However, does this require a diagnosis of CKD? Or is the knowledge of having reduced kidney function sufficient for the patient to understand their risks? This needs to be further explored with a trial of different labels for mild decline in kidney function in older patients. The patient perspective was not sought as part of this research project. It will be very important to obtain the patient viewpoint on use of different labels for CKD as part of future research on what is the most appropriate labelling of patients.

In conclusion, GPs are persistent in using descriptions of 'reduced kidney function' to explain CKD to their patients, either in parallel with the CKD label or instead of it. GPs had concerns about the words 'chronic' and 'disease' and used different terminology to explain these words to patients when diagnosing them with CKD. These concerns were particularly around older patients with a mild decrease in their kidney function. Use of an alternative label that denotes the level of risk to older patients, without creating unnecessary concern needs to be explored, as well as increased recognition of the normal reduction in kidney function with ageing.

## Data

The data comprises de-identified interview transcripts, and is available on reasonable request by emailing researchdata@bond.edu.au.
